# Advantages of robot-assisted PKP under local anesthesia in the treatment of OVCF: a retrospective, non-randomized, controlled, clinical study

**DOI:** 10.3389/fsurg.2024.1445461

**Published:** 2024-08-08

**Authors:** Han Xue, Wei Liu, Ruochen Li, Fengxu Xiao, Zheyue Zhu, Guangwei Wu, Chen Zhang

**Affiliations:** ^1^ Department of Orthopaedic Center, The Second Affiliated Hospital of Xi’an Jiaotong University, Xi’an, China; ^2^ Department of Sports and Joint Surgery, Xian Yang Central Hospital, Xian Yang, China; ^3^ Department of Ultrasound, The Second Affiliated Hospital of Xi’an Jiaotong University, Xi’an, China

**Keywords:** osteoporotic vertebral compression fracture, pedicle puncture, robot-assisted, percutaneous kyphoplasty, local anesthesia

## Abstract

**Background:**

Robot-assisted technology has been widely used in orthopedic surgery, which can provide surgeons with higher accuracy and reduce radiation exposure. In spinal surgery, robots are often used to assist pedicle screw implantation, while there are relatively few studies on robot-assisted percutaneous kyphoplasty (PKP) under local anesthesia.

**Methods:**

A total of 96 patients with single-segment OVCF who met the inclusion criteria were included in this study. Fifty-six patients underwent robot-assisted PKP and forty patients underwent conventional PKP by the same group of surgeons. Collect the relevant parameters.

**Results:**

The puncture time and fluoroscopy times during puncture in the robot group were significantly less than those in the manual group (*P* < 0.001). The success rate of first puncture in the robot group was 92.5%.

**Conclusions:**

PKP under local anesthesia assisted by the new spinal surgical robot effectively reduces the patient's intraoperative discomfort and has a low learning curve.

## 1 Introduction

Osteoporosis is defined as a systemic bone disease characterized by low bone mass and deterioration of bone tissue microstructure, resulting in bone fragility and increased fracture susceptibility (1). With the aging of the population, osteoporosis has become an important public health problem. In 2016, the prevalence of osteoporosis in the elderly over 60 years old in China was 36%, the incidence was 23% in men and 49% in women (2, 3). Osteoporotic fracture (or fragility fracture) is a serious consequence of osteoporosis, which refers to fractures that occur after minor trauma. Osteoporotic fracture is one of the main causes of disability and death in elderly patients, which is harmful to health and can lead to persistent pain, deformity, weight loss, depression, decreased quality of life and even death. Among them, osteoporotic vertebral compression fracture (OVCF) is the most common (4). OVCF affects about 1.4 million people worldwide every year, and the incidence is higher than 20% in the elderly (5). Although conservative treatment still provides some effective symptom management, infectious diseases of the respiratory system and urinary system, as well as kyphosis, are common problems after OVCF (6). Percutaneous kyphoplasty (PKP), whose effectiveness has been confirmed in a number of systematic reviews and clinical trials as well as its safety, is currently an effective and safe method for the treatment of OVCFs in the elderly, especially for patients with fracture less than 6 weeks and severe pain (4, 5, 7). It can quickly relieve pain, prevent vertebral collapse, prevent bed rest or reduce related complications during bed rest, restore self-care ability and reduce mortality (8).

PKP is generally under local anesthesia, which has higher requirements for patient cooperation, accuracy of puncture, and number of puncture adjustments (9–11). Traditional pedicle access requires fluoroscopic guidance to improve accuracy and safety. Although the operation is simple, it has the following drawbacks: the surgeon usually needs to repeat x-ray examination to determine the safe puncture path, and the radiation exposure to the patient and the doctor is large. It is not intuitive for doctors to judge only by fluoroscopy images, which requires high proficiency of the operator. Moreover, due to the deviation of fluoroscopy, the safety and accuracy of puncture may be affected (12, 13). With the continuous innovation and development of robot-assisted technology, orthopedic surgical robots have become the core of promoting accurate and minimally invasive orthopedic treatment (14). The meta-analysis of Li et al. (15), which included 152 clinical studies, described the types of robotic surgical information, and showed that robot-assisted surgery was mainly used in joint replacement and spinal surgery, which required high precision. In spinal surgery, robot-assisted surgery was mainly used to assist pedicle channel establishment and pedicle screw implantation (16, 17). At present, the main domestic spinal surgery robot platforms include ROSA One, Mazor X and TiRobot. The patients need to be under general anesthesia to complete the pedicle channel construction and pedicle screw implantation, which are rarely used in the operation under local anesthesia. This limits the application of commonly used robotic systems in PKP under local anesthesia, and there are few relevant studies.

The new spinal surgical robot system used in this study uses a direct visual positioning method to realize direct navigation for pedicle feature image recognition. Different from the robot currently using binocular vision positioning method, there is no need to establish a navigation coordinate system under apnea, which avoids the influence of invasive operation and position micromovement on image positioning, and makes surgery under local anesthesia possible. By evaluating the surgical indicators and clinical effect indicators of the robot-assisted surgery, and comparing the results with those of manual operation and other robot-assisted surgery, we explored the advantages of the new spinal surgery robot.

## 2 Materials and methods

### 2.1 Equipment and principle

**Figure 1:**
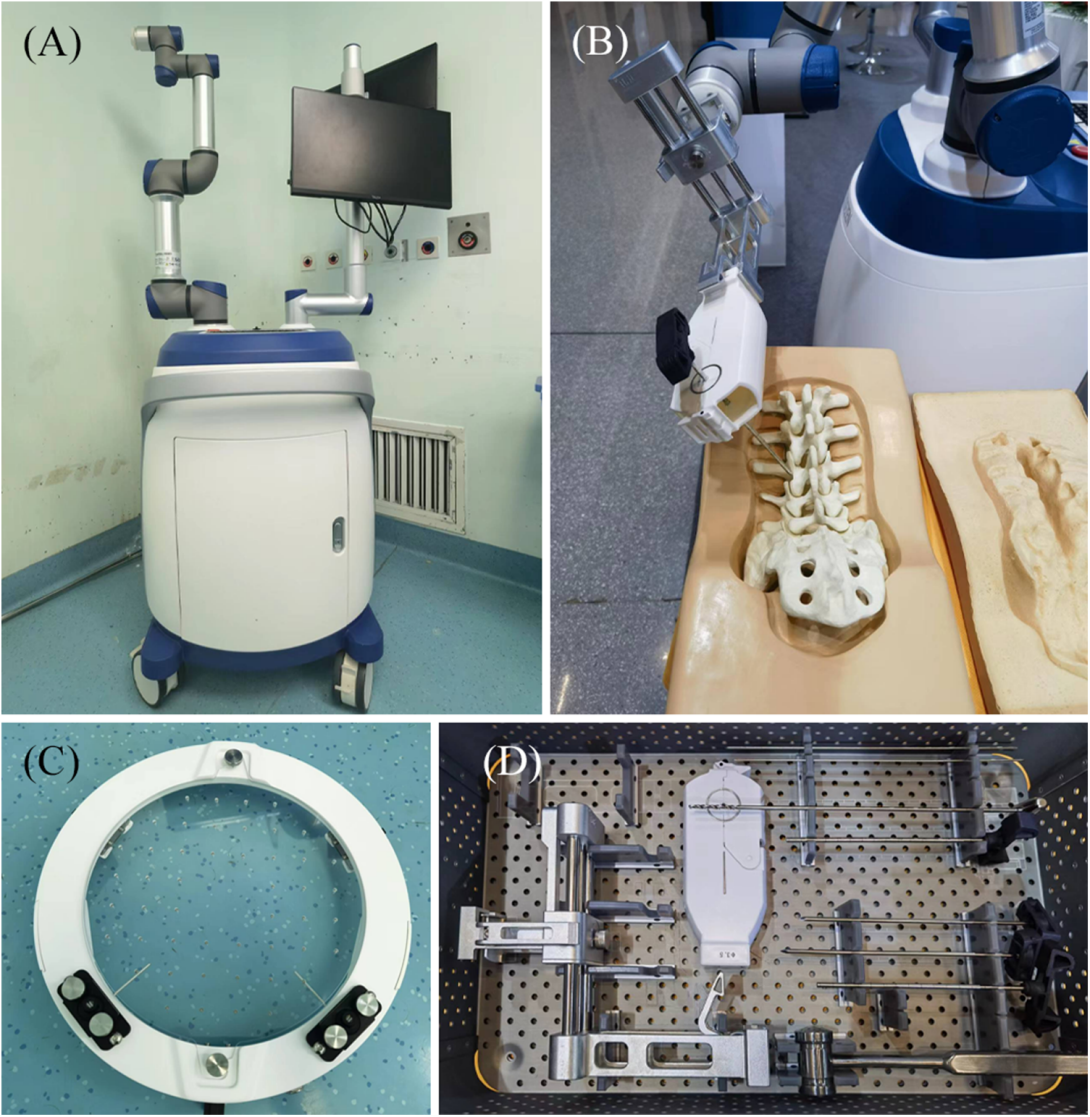
Zuohang 300 robot system (ZOEZEN, China). (**A**) Zuohang 300 spinal robot mainframe. Includes control workstation, monitor, robot arm and software system. (**B**) Simulation of robot working state. (**C,D**) Special equipment for the Zuohang 300 spinal robot. Including image correction and laser positioning device, path positioning frame and double concentric rings positioning sleeve, and surgical instruments.

The Zuohang 300 robot system (ZOEZEN, China) for minimally invasive spinal surgery was used in this study (Figure 1).

**Figure 2:**
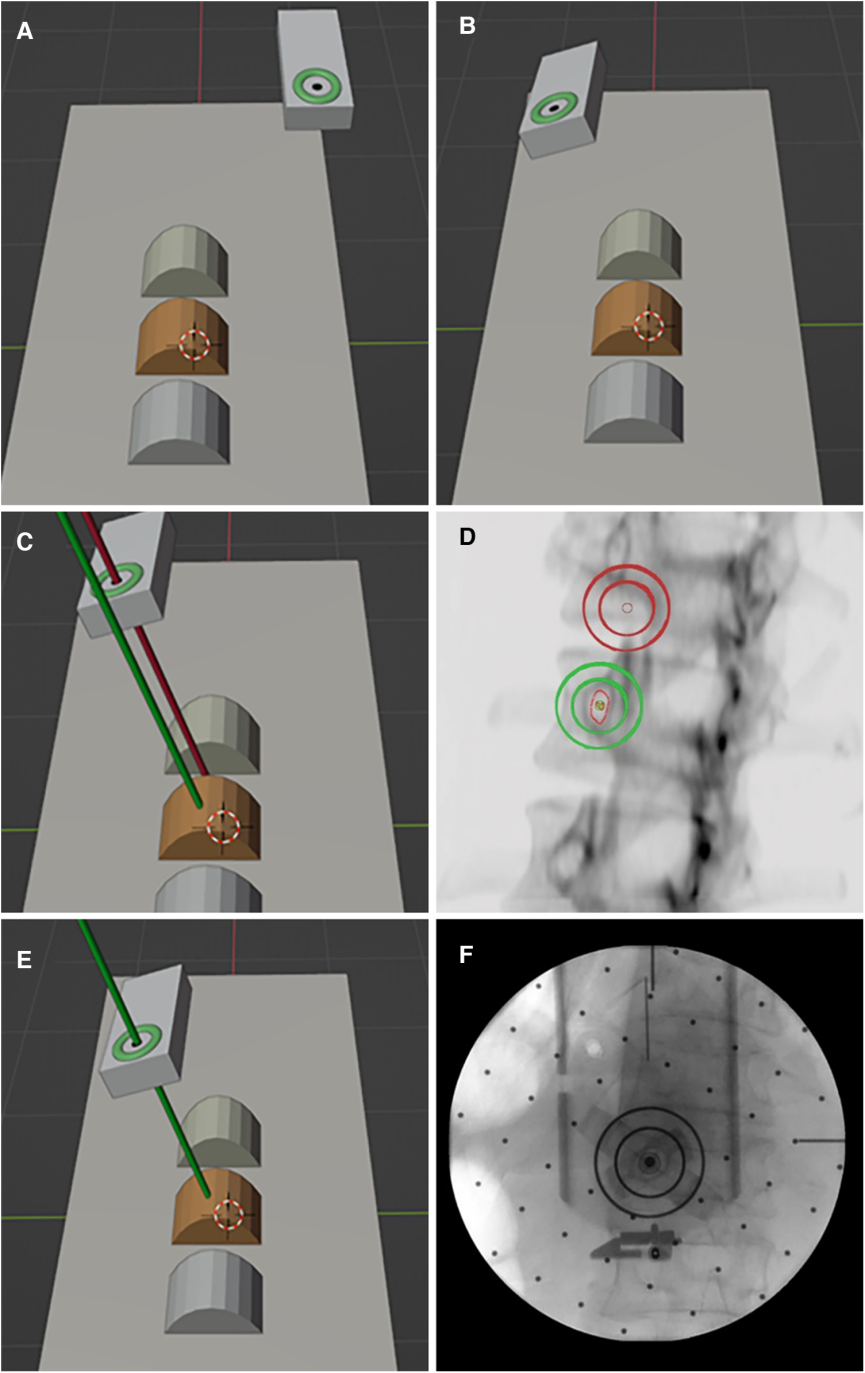
Principle of pedicle puncture in the robot navigation system. (**A,B**) The double ring moves near the target position and adjusts to the preset angle. (**C,D**) Pedicle axial fluoroscopy. (**E,F**) The double ring was moved to the target position and verified by fluoroscopy.

Based on the pinh pedicle ole imaging principle and the safe pedicle puncture principle of “the axis of the and the axis of the pedicle puncture needle coincide”, the robot navigation system adopts the intuitive imaging method of double concentric ring positioning, calculates the position and relationship between the surgical path (pedicle channel) and the end of the robotic arm in the characteristic image space, and finally determines the position and direction of the pedicle channel (Figure 2).

### 2.2 Clinical treatment

#### 2.2.1 Grouping and inclusion and exclusion criteria

A retrospective analysis was conducted on the clinical data of patients who underwent percutaneous kyphoplasty (PKP) surgery at our orthopaedic center between September 2021 and March 2023. The specific inclusion and exclusion criteria were as follows.

Inclusion criteria: (1) Age: male >60 years old, female >55 years old; (2) vertebral bone mineral density measurement confirmed OP; (3) Imaging showed a single-segment fresh fracture of the vertebral body, with an intact posterior wall, and no intraspinal space-occupying lesion or spinal cord compression; (4) no lower extremity neurological symptoms.
Exclusion criteria: (1) vertebral tumors and pathological fractures; (2) vertebral burst fracture; (3) old thoracolumbar fractures; (4) poor physical condition or inability to tolerate surgery.

Based on the above inclusion and exclusion criteria, a total of 96 patients with single-segment osteoporotic vertebral compression fractures were included in the study. The patients were divided into two groups according to the surgical approach: a traditional manual PKP group (manual group, *n* = 56) and a robot-assisted PKP group (robot group, *n* = 40). Due to the limited registration of the robot, it can only assist the lower thoracic and lumbar spine surgery. In addition to the surgical methods, the intraoperative instruments and implants (China Resources Corporation, China), postoperative care and rehabilitation, and postoperative follow-up were the same in the two groups. All operations were performed by the same experienced surgeon. The study was approved by the Ethics Committee of our hospital. All patients provided written informed consent and agreed to the release of images involving their bodies.

#### 2.2.2 Surgical methods

1. Manual group

Preoperative preparation: The patient's imaging data were observed to estimate the position and Angle of puncture.

Intraoperative procedures: (1) The patient was placed in the prone position, and the injured vertebrae were confirmed and marked by anteroposterior and lateral fluoroscopy. (2) routine disinfecting and draping. (3) The position and Angle of the needle were estimated according to the marking points, local infiltration anesthesia was performed, the skin was cut 3 mm, and the unilateral needle was selected. The puncture needle was fixed on the bone surface by gently tapping the needle with a bone hammer, and the needle entry point was adjusted to the appropriate position by fluoroscopy in the anteroposterior and lateral view. The puncture was performed through the pedicle and verified by C-arm fluoroscopy. The anteroposterior and lateral images were compared to ensure that the puncture needle was located in the pedicle. If the puncture needle is found to be skewed, it should be adjusted in time until the fluoroscopy is detected in a good position.

The puncture needle was further penetrated into the mid-posterior third of the vertebral body. The sleeve core was extracted, and the reamer bit was implanted and drilled to 3 mm from the anterior edge of the vertebral body. Anteroposterior and lateral fluoroscopy was performed to confirm that the bit was in the safe range. After withdrawing the drill, a balloon dilator was implanted into the mid-anterior third of the vertebral body, and the developer was injected into the balloon under intermittent fluoroscopy monitoring. After satisfactory distraction, the developer was withdrawn and the balloon was withdrawn. The bone cement was pushed in under intermittent fluoroscopic monitoring, and the filling condition was observed while pushing to ensure that the bone cement did not leak until the dispersion of the bone cement was satisfactory. A puncture needle was inserted, and the needle was pulled out after the cement hardened. Sterile suture and bandage were used, and the procedure was completed.

2. Robot group

**Figure 3:**
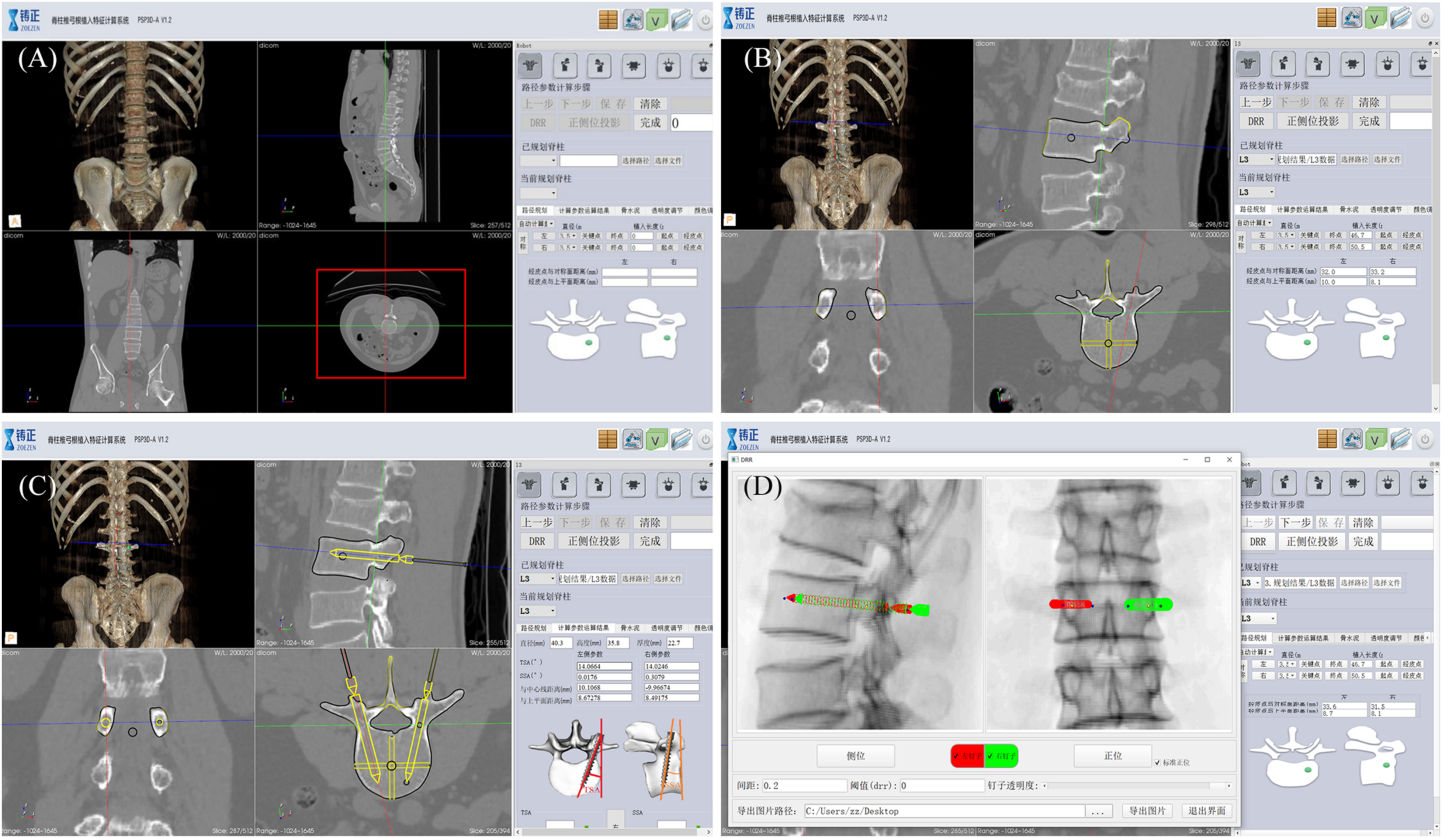
Robot-assisted preoperative planning process. (**A**) Import the data and select the target vertebra. (**B**) Measure vertebral parameters, calibrate key points, and generate channels. (**C**) The path planning parameters were calculated and the data results were obtained. (**D**) Generate and preview the DRR (digital reconstruction radiology image), and save the data.

Preoperative planning: Preoperative CT examination of the surgical site was completed, and the engineer processed the CT data and communicated with the doctor about the feasibility of 3D planning of the implantation path (Figure 3).

**Figure 4:**
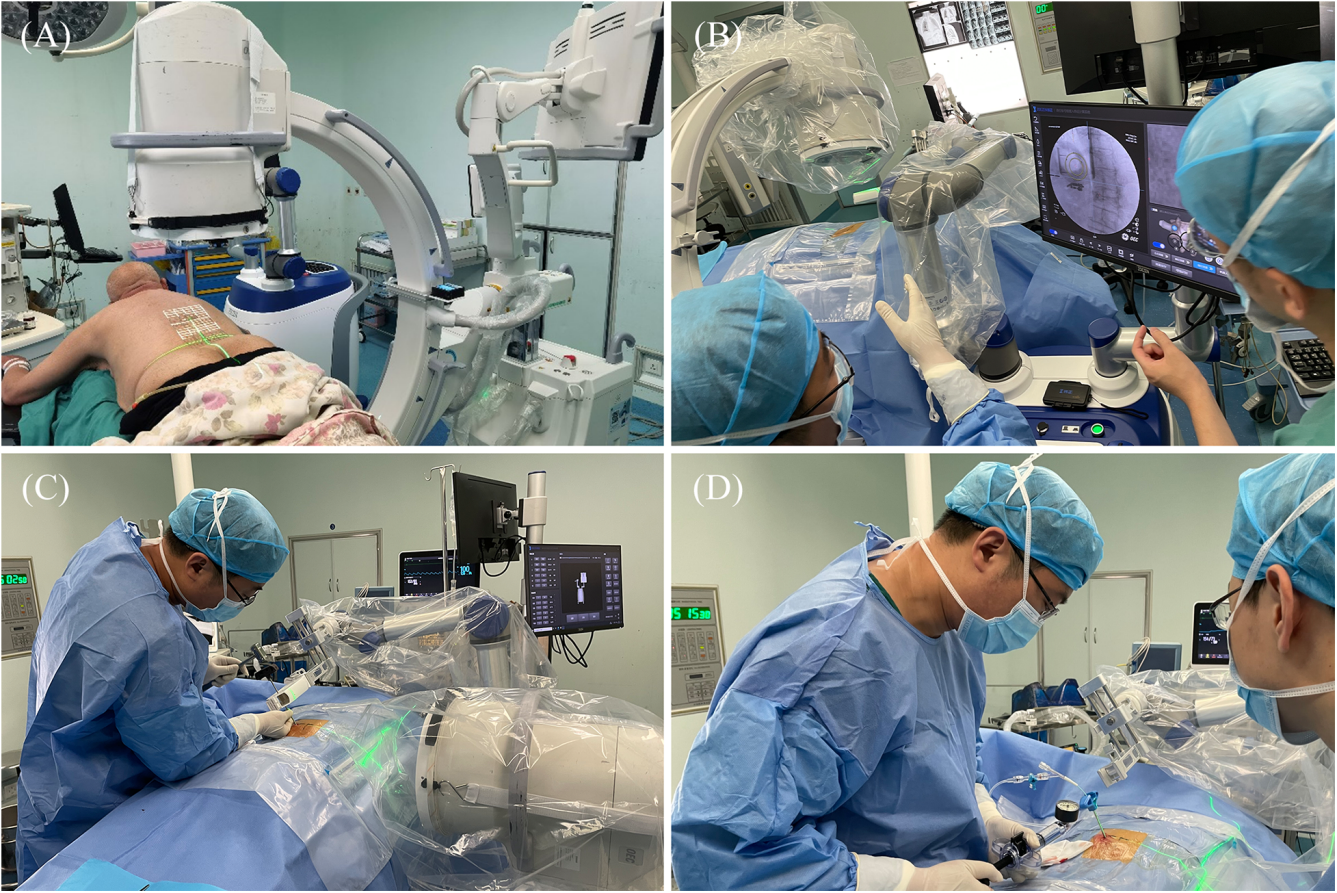
Intraoperative procedure of robot group. (**A**) Anteroposterior and lateral fluoroscopy was used to mark the injured vertebrae and complete the registration. (**B**) The final position was confirmed by intraoperative navigation software. (**C**) The needle was inserted along the planned path of the robot. (**D**) When the access was satisfactory, bone cement was injected.

**Figure 5:**
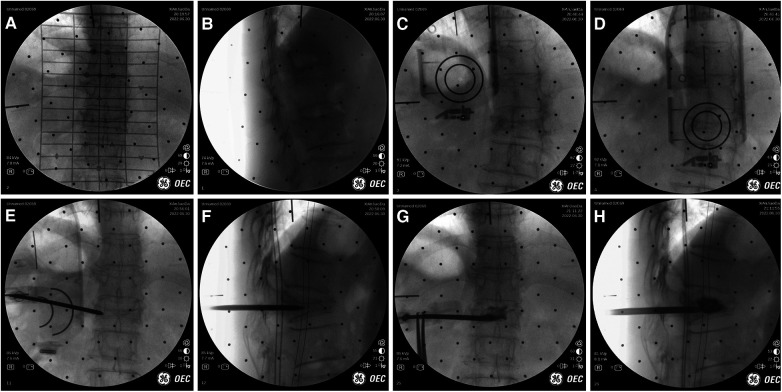
Intraoperative images of robot-assisted surgery. (**A,B**) Anteroposterior and lateral fluoroscopy was used to mark the injured vertebrae. (**C,D**) The position and direction of needle insertion were determined with the assistance of robot. (**E,F**) The pedicle path was established along the planned path. (**G,H**) Fluoroscopy into the bone cement.

Intraoperative procedures: (1) Patients were placed in the prone position, and standard anteroposterior and lateral fluoroscopy was performed first. A level was installed on the rotation axis of the C-arm to correct the Angle, and an image corrector and a laser emission device were installed on the launching end of the C-arm. The image corrector was used to eliminate the image distortion caused by fluoroscopy, and the laser emission device was used to locate the pedicle axis during the subsequent fluoroscopy. The anteroposterior and lateral fluoroscopic images were fitted to the DRR in the software to complete the contour-based 2D-3D registration and calibrate the preoperative planning data. At the same time, the injured vertebrae were confirmed and marked by the positioning plate. (2) Routine disinfecting and draping. (3) The control workstation controlled the mechanical arm to rotate the end of the double ring to the preset Angle, and then controlled the mechanical arm to translate the double ring to the position approximately aligned with the target pedicle. The C-arm was adjusted so that the laser passed through the central hole of the double ring and fluoroscopy verification was performed to confirm that the double ring was concentric, and the axis of the double ring was parallel to the axis of the pedicle. The center position of the double ring on the fluoroscopy image was compared with the target pedicle needle insertion position by the intraoperative navigation software, and the required adjustment distance was calculated according to the position parameters. The control workstation controlled the robotic arm to automatically adjust the center of the double ring to the target pedicle needle insertion position, and then the pedicle axial fluoroscopy was performed again to confirm that the center of the double ring fell in the target position. Under local infiltration anesthesia, the skin was cut 3 mm, and unilateral needle insertion was selected. The puncture needle was inserted along the central hole of the double ring until it reached the bone surface. The bone hammer was used to gently tap the puncture needle on the bone surface, and the axial fluoroscopy was used to verify that the puncture needle was projected into the central hole of the double ring and did not exceed the boundary of the central hole. Puncture through the pedicle was verified under C-arm fluoroscopy. The follow-up operation was the same as that of the manual group (Figures 4, 5).

#### 2.2.3 Postoperative nursing and rehabilitation

Vital signs were monitored within 6 h after surgery. After operation, the patients were kept in bed, and functional exercise of both lower limbs was performed while lying in bed. One day after operation, the patients got out of bed with orthosis, and the anteroposterior and lateral x-ray films were reviewed. Systemic anti-osteoporosis drugs were used during perioperative period and after discharge.

### 2.3 Data collection

All clinical data were collected and analyzed by dedicated research staff.

#### 2.3.1 Basic information

The basic data of each group were recorded, such as gender, age, body mass index (BMI), bone mineral density (BMD), injured vertebrae, and time from injury to surgery.

#### 2.3.2 Surgical indicators

1. The total operation time and the time of preparation process, puncture process, and bone cement injection and other subsequent processes.
  Total operation time was defined. The total operation time was calculated from the starting time when the patient's position was ready and the end time when the patient's wound was dressed.
  Puncture procedure time was defined. Manual group: after local anesthesia, the time from the start of puncture to the puncture to the target position was calculated; Robot group: After the registration of the robot, the time from the start of robot-assisted positioning to the completion of pedicle puncture was calculated.
2. The total number of fluoroscopy and the number of fluoroscopy during positioning, puncture, subsequent needle insertion and cement injection.
3. The number of punctures and the success rate of first puncture
4. blood loss
5. operation-related complications

#### 2.3.3 Clinical effect indicators

1. Imaging

HFV (the height of the most obvious compression of the fractured vertebral body on the lateral x-ray film) and VKA (the Angle between the upper and lower endplates of the fractured vertebral body on the lateral x-ray film) were measured on the preoperative and postoperative vertebral radiographs.

2. Scales and scores

The visual analogue scale (VAS) and Oswestry disability index (ODI) were recorded at admission, 1 day and 3 months after operation to evaluate the postoperative recovery.

### 2.4 Statistical analysis

SPSS 25 software package was used for statistical analysis. The measurement data were in accordance with normal distribution and were described by mean ± standard deviation. The statistical inference of categorical data was performed by chi-square test, and the independent sample *t*-test was used for comparison between groups. The significance level *α* = 0.05 was set, and *p* ≤ 0.05 was considered statistically significant.

## 3 Results

All robot-assisted surgeries were performed by the same experienced doctor. Robot-assisted surgeries required cooperation from multiple parties and the previous operations needed to be run-in, so the three patients in the early stage were not included in research and statistics, which was reflected in the analysis of learning curve.

### 3.1 Analysis of patient data

A total of 92 patients with single-segment osteoporotic vertebral compression fracture were included in the study. All patients were diagnosed as osteoporosis by DXA. Based on χ^2^ test and *t*-test, there was no significant difference between the robot group (*n* = 40) and the manual group (*n* = 52) in the basic data such as gender, age, BMI, BMD, distribution of injured vertebrae, and time from injury to operation (*p* > 0.05) (Table 1).

**Table 1:** Basic information of the two groups of patients.

Grouping	Gender		Injured vertebral body		Time between injury and operation
	Male	Female	T8–T12	L1–L5	
Manual group (*n* = 52)	14	38	30	22	4.92 ± 2.33
Robot group (*n* = 40)	9	31	21	19	4.30 ± 2.90
χ^2^/*t*	0.059		0.247		1.155
*p*	0.808		0.619		0.251
Grouping	Age		BMI		BMD
Manual group (*n* = 52)	72.12 ± 9.30		23.45 ± 1.96		3.10 ± 0.49
Robot group (*n* = 40)	77.63 ± 7.98		24.13 ± 1.69		3.19 ± 0.47
χ^2^/*t*	1.906		1.738		0.845
*p*	0.060		0.086		0.400

### 3.2 Surgical data analysis

The operation time was compared between the two groups. The preparation time of the robot group (14.96 ± 1.72 min) was longer than that of the manual group (9.94 ± 1.82 min), the difference was statistically significant (*p* = 0.000). The puncture time of the robot group (5.46 ± 1.97 min) was significantly shorter than that of the manual group (12.02 ± 2.87 min), and the difference was statistically significant (*p* = 0.000). There was no significant difference in the total operation time between the robot group (39.98 ± 3.81 min) and the manual group (41.15 ± 3.92 min) (*p* > 0.05).

Comparison of radiation exposure between the two groups. The fluoroscopy times of the robot group was significantly less than that of the manual group (4.25 ± 1.50 vs. 9.50 ± 2.48, *p* = 0.000). The total number of fluoroscopy in the robot group (28.45 ± 3.34) was less than that in the manual group (33.35 ± 3.87), and the difference was statistically significant (*p* = 0.000). The surgeon was far away from the operation during fluoroscopy, and there was no radiation exposure.

The number of punctures in the robot group was significantly less than that in the manual group (1.35 ± 0.98 vs. 5.08 ± 1.40, *p* = 0.000). The success rate of first puncture in the robot group was 92.5%. There was a significant difference in intraoperative blood loss between the two groups (robot group 5.50 ± 1.80 ml, manual group 6.85 ± 2.32 ml, *p* < 0.05). There were 2 cases of bone cement leakage in the robot group and 5 cases in the manual group, and there were no clinical manifestations such as neurological symptoms. There were no major complications such as bone cement leakage embolism, nerve or vascular injury, infection, secondary vertebral fracture, or death in the two groups after operation and up to the last follow-up time (Table 2).

**Table 2:** Data of surgical indicators of the two groups.

Grouping	Procedure Time (min)			
	Preparation	Puncture	Follow-up	Total time
Manual group (*n* = 52)	9.94 ± 1.82	12.02 ± 2.87	19.19 ± 2.15	41.15 ± 3.92
Robot group (*n* = 40)	14.96 ± 1.72	5.46 ± 1.97	19.57 ± 2.08	39.98 ± 3.81
*t*	13.47	12.40	0.850	1.431
*p*	0.000	0.000	0.398	0.156
Grouping	Number of fluoroscopy (times)			
	Positioning	Puncture	Follow-up	Total number of fluoroscopy
Manual group (*n* = 52)	4.73 ± 1.30	9.50 ± 2.48	19.12 ± 2.69	33.35 ± 3.87
Robot group (*n* = 40)	4.70 ± 1.02	4.25 ± 1.50	19.50 ± 2.36	28.45 ± 3.34
*t*	0.123	11.83	0.716	6.382
*p*	0.902	0.000	0.476	0.000
Grouping	Number of punctures (times)	Success rate of one puncture	Amount of blood loss (ml)	Number of patients with complications
Manual group (*n* = 52)	5.08 ± 1.40	0	6.85 ± 2.32	5
Robot group (*n* = 40)	1.35 ± 0.98	92.5%	5.50 ± 1.80	2
*t*	14.37		3.033	
*p*	0.000		0.003	

### 3.3 Analysis of clinical effect indicators

The imaging indicators of the two groups: HFV and VKA in the two groups were improved. There were significant differences in HFV and VKA between the two groups before and after surgery (*p* < 0.05), and there was no significant difference in the change values of HFV and VKA between the two groups before and after surgery (*p* > 0.05).

Scales and scores of the two groups: the symptoms of low back pain were significantly relieved in the two groups. The VAS and ODI scores at 1 day after operation were significantly better than those before operation, and further improved at 3 months after operation. There were significant differences in VAS and ODI scores before and after operation in the two groups within the group, but there were no significant differences in VAS and ODI scores between the two groups before operation, 1 day and 3 months after operation (*p* > 0.05) (Table 3).

**Table 3:** Clinical effect index data of the two groups of patients.

Grouping	HFV (cm)		
	Before surgery	After surgery	Changing values
Manual group (*n* = 52)	14.12 ± 1.90	20.84 ± 2.57	6.72 ± 1.77
Robot group (*n* = 40)	15.63 ± 2.24	23.01 ± 2.52	7.38 ± 2.52
*t*	3.485	4.406	1.476
*p*	0.001	0.000	0.143
Grouping	VKA (°)		
	Before surgery	After surgery	Changing values
Manual group (*n* = 52)	17.85 ± 2.70	10.38 ± 4.01	7.47 ± 3.26
Robot group (*n* = 40)	16.14 ± 2.45	8.63 ± 2.41	7.51 ± 2.75
*t*	3.137	2.437	0.055
*p*	0.002	0.017	0.956
Grouping	VAS		
	Before surgery	1 day after surgery	3 months after surgery
Manual group (*n* = 52)	6.39 ± 0.89	1.96 ± 0.59	1.08 ± 0.48
Robot group (*n* = 40)	6.40 ± 0.98	1.75 ± 0.44	1.10 ± 0.44
*t*	0.079	1.892	0.237
*p*	0.938	0.062	0.813
Grouping	ODI		
	Before surgery	1 day after surgery	3 months after surgery
Manual group (*n* = 52)	81.22 ± 5.41	40.60 ± 5.95	19.93 ± 6.21
Robot group (*n* = 40)	81.56 ± 6.69	38.00 ± 6.40	19.90 ± 5.64
*t*	0.271	1.691	0.027
*p*	0.787	0.094	0.979

### 3.4 Learning curve

**Figure 6:**
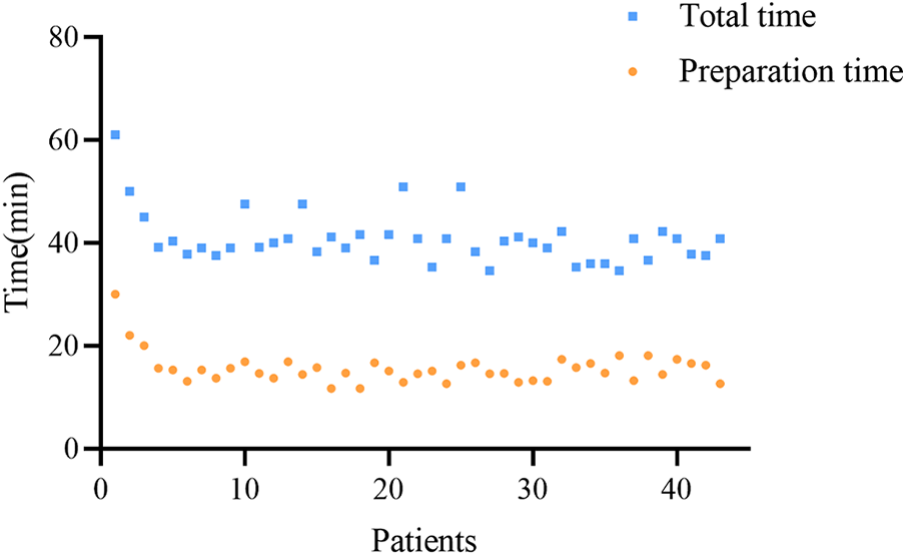
Learning curve of robot-assisted pedicle puncture.

For the robot-assisted pedicle puncture technique, after performing 3 procedures, the robot-assisted pedicle puncture preparation time and the total operation time decreased. In addition, the surgeon can quickly master the technique over time, and the preparation time and total operation time are stable in subsequent procedures (Figure 6).

## 4 Discussion

Traditional manual procedures rely on repeated fluoroscopy monitoring, which exposes patients or surgeons to radiation for a long time. The non-standard fluoroscopy Angle and distortion of fluoroscopy image lead to large errors in the process of puncture. The puncture technique depends on the surgeon's mastery of the anatomical structure, which is difficult and requires a long learning period, especially for junior residents, who need a relatively long learning process to master the technique. Robot-assisted surgery can reduce the number of punctures and radiation exposure by its accuracy. A good perspective direction can be obtained through a specific device and algorithm to overcome the image distortion caused by the perspective and reduce the puncture error. It also reduces the learning curve of pedicle puncture technique (18).

However, during the preliminary study, we observed significant variability in the results of existing studies on robot-assisted technology. This variability was attributed to differences in operator proficiency, equipment used, and surgical methods, making the horizontal comparisons between different studies challenging. Additionally, the meta-analyses did not strictly define the variations in equipment and surgical techniques, rendering it difficult to draw definitive conclusions regarding the value of robot-assisted techniques (18, 19).

Surgical time is one of the commonly used metrics to assess the value of the application of surgical techniques. However, previous studies have reported conflicting results regarding surgical time. In previous studies using the Tirobot to assist PKP, Wang et al. (20) reported comparable operative times between the robot and manual groups, while Jin et al. (21) and Lin et al. (22) reported shorter operative times in the robot group, and Li et al. (23) and Yuan et al. (24) reported longer operative times in the robot group. Conversely, in studies utilizing the ZOEZEN robot, Shi et al. (25) and Tan et al. (26) demonstrated significantly shorter total operative and puncture times in the robot group compared to the freehand group. In the present study, although no significant difference was observed in the total operative time between the two groups, the preparation time was longer in the robot group compared to the freehand group, while the puncture time was significantly shorter in the robot group. The surgeon's experience and the equipment and techniques used were identified as the main factors influencing the operative time. During the preoperative preparation phase, the robot group required more time for setting up and debugging the surgical robot, thereby extending the total surgical time. However, during the surgical phase, the robot group was significantly faster than the manual group due to its precision, which compensated for the extended time required during the preoperative preparation.

In this study, we used the number of fluoroscopies (i.e., fluoroscopy frequency) as an important parameter for assessing radiation exposure time. Our results indicated no significant difference in fluoroscopy frequency between the robot and manual groups during the preoperative positioning and subsequent cement injection phases. However, during the puncture phase, the fluoroscopy frequency in the robot group was significantly lower than that in the manual group. Eventually, the total fluoroscopy frequency of the robot group was lower than that of the manual group. Similar findings were reported by Shi et al. (25) Based on our postoperative analysis, we concluded the following: (1) robot-assisted technology facilitates accurate and rapid surgical path planning, effectively reducing fluoroscopy frequency during the puncture process; (2) localization of the injured vertebral body and the cement injection process still rely on the surgeon's experience. While current robot-assisted technology aids in timely and precise intraoperative adjustments, it does not fully encompass the entire surgical procedure. Additionally, variations in surgical robots may contribute to differences in fluoroscopy frequency.

Intraoperative blood loss and cement leakage are often used to comprehensively assess the safety of the procedure. In this study, although there was a statistically significant difference in the amount of bleeding between the two groups, the clinical relevance may be minimal given that PKP is a minimally invasive procedure. Additionally, regarding cement leakage, most studies have shown a lower rate in the robot group (22, 27, 28). This is likely due to the more precise puncture facilitated by preoperative scanning and planning methods in the robot group, significantly reducing the risk of vertebral bone and pedicle injuries. In contrast, the manual group, which may require multiple punctures, further compromises vertebral body integrity. In this study, cement leakage occurred in five patients in the manual group compared to two patients in the robot group.

Pain relief is often the primary concern for patients. The results of this study showed no significant difference in postoperative VAS scores between the two groups. However, Jin et al. (21) reported lower short-term postoperative VAS scores in the robot group compared to the manual group. This discrepancy may be attributed to the fact that conventional fluoroscopy-assisted pedicle puncture often requires repeated adjustments of the puncture angle to ensure the needle does not breach the pedicle wall. Such adjustments can exacerbate damage to muscles, fascia, and other soft tissues, resulting in suboptimal postoperative pain relief. In contrast, robot navigation offers greater accuracy and reduces muscle irritation. Additionally, robot-assisted implants can be positioned more precisely near the midline or fracture line, allowing the cement to be diffusely distributed along the midline of the vertebral body or fracture region, which may enhance pain relief (29–31).

Postoperative imaging is essential for evaluating outcomes. Yuan et al. (24) demonstrated that the postoperative improvement in HFV and LKA was significantly greater in the robot group compared to the manual group. This enhancement is likely due to the robot assistance's ability to more effectively open the vertebral body for repositioning and to accurately position the balloon. In contrast, Guo et al. (32) found no significant difference in the improvement of spinal height and kyphosis between the robot and manual groups. The results of the present study are consistent with Guo et al.'s findings (32). In this regard, we believe that there may be no difference in imaging performance if skilled manual manipulation is used to accurately place the balloon in the middle of the vertebral body or at the site of the most severe fracture collapse.

Similar to the learning curve for freehand fluoroscopy in pedicle puncture, the robot-assisted pedicle puncture technique also has its own learning curve. Surgeons need to understand the characteristics of the robot system and master the procedure, including effectively integrating the robotic operating system into the overall surgical workflow. Previous studies have reported that the learning curve for fluoroscopic pedicle screw placement ranges from 20 to 50 procedures, with a minimum of 25 procedures required to achieve proficiency in this technique (33, 34). Kam et al. (34) demonstrated that robot-assisted pedicle screw placement involves a short and negligible learning curve by evaluating the accuracy of screw placement, time to completion, radiation exposure, and complication rates. In this study, the robot-assisted pedicle puncture preparation time and total procedure time decreased and gradually stabilized after three procedures were performed. Following the initial learning cycle of positioning, successful positioning, and successful puncture, the surgical technique was standardized, providing effective assistance to less experienced surgeons.

One of the main advantages of the robot-assisted technology used in this study is its ability to support patients undergoing surgical procedures under local anesthesia. Unlike other robot platforms such as ROSA, MAZOR, and Ti-Robot, which utilize 3D imaging principles (35–39), the ZOEZEN robot employs intuitive image navigation to provide a simple-to-operate puncture path. This system eliminates the need for an additional tracking reference frame and does not require consideration of the deflection and displacement of the reference system relative to the vertebral body. Intraoperative x-ray images serve as the basis for robot navigation and safety confirmation. During the procedure, the surgeon can visually confirm whether the protrusion formed by the puncture cannula is within the contour of the pedicle and ensure that the guide needle safely enters the vertebral body, thereby enhancing patient safety. This also means that minor changes in the patient's position during the operation will have minimal impact on the accuracy of the robot's navigation and positioning. Therefore, unlike currently applied robotic systems that require the patient to be under general anesthesia, this robot (ZOEZEN) can be applied to surgery under local anesthesia.

During the clinical application of this technology, we encountered a series of challenges. Firstly, some patients have a low pain threshold, and local anesthesia alone was insufficient to meet their analgesic needs. As a result, these patients often changed their positions due to pain during the operation. To address this, we ensured the effectiveness of local anesthesia and administered intravenous analgesic drugs. This multimodal analgesia approach improved patient tolerance and adherence, allowing the surgery to proceed smoothly under local anesthesia. If necessary, we also adjusted the surgical treatment plan. For patients with hypertension, the prone surgical position and pain stimulation while awake can cause significant blood pressure fluctuations, increasing surgical risk. Therefore, in addition to oral antihypertensive drugs, we temporarily administered intravenous antihypertensive drugs intraoperatively to enhance surgical safety. Robot-assisted surgery under local anesthesia is not recommended for patients with chronically unstable preoperative blood pressure control. The ability to maintain effective communication during surgery is also one of the highlights of surgery under local anesthesia. Most of the patients have fear and anxiety about the surgical process. The best way to calm the patients is to maintain effective communication with them. Timely understanding of the patient's feelings, informing the patient of the surgical process and appropriate verbal appeasement are all conducive to relieving the patient's nervousness and anxiety, which can help ensure the smooth progress of the surgery.

The robot used in this study uses visual positioning instead of determining spatial positioning coordinates, which cannot be widely used as other integrated platform robots. More extensive applications are also under further development, such as the establishment of bone tunnel in anterior cruciate ligament reconstruction under visual imaging. Accordingly, robotic surgery requires additional staff, additional preparation time, and teamwork, resulting in a more stringent choice of surgical timing that may not be appropriate for patients in urgent need of surgery. The establishment of pedicle access has a great relationship with the proficiency of the surgeon. This study was completed by experienced surgeons, and the more difficult operations during the operation are easy to be ignored. Therefore, it is of greater significance for junior surgeons. At the same time, the cost of the robot should be considered, and the robot is relatively inexpensive to use. It has far-reaching significance in popularizing minimally invasive spinal surgery in primary hospitals, as well as escorting primary medical care.

There are several limitations to acknowledge in this study. This was a retrospective, non-randomized, controlled, clinical study. Case selection bias may have confounded the study data. The longest follow-up time in this study was 3 months after surgery. Also, the inclusion of only single segments and unilateral punctures is one of the limitations of this study. In further studies, we will continue to conduct prospective, multicentre studies to explore factors affecting clinical outcomes through longer follow-up times to assess the potential benefits and long-term prognosis of robot-assisted surgery.

## 5 Conclusions

The results of different robot-assisted PKP studies are different. Compared with the traditional manual operation, the main advantages of the new spinal surgery robot-assisted PKP under local anesthesia in this study are to reduce the puncture time and improve patient satisfaction. The fluoroscopy times during the puncture process were reduced to reduce the radiation exposure of patients. It also reduces the number of puncture attempts, patient pain and possible complications caused by multiple punctures. Because this study was performed by an experienced surgeon, there was no statistical difference in clinical efficacy indicators between the two groups, and a longer follow-up may be needed to explore the long-term benefits. Therefore, robot-assisted surgery is more meaningful for novice surgeons, who can quickly master the technology through a shorter learning curve. At the same time, however, robot-assisted surgery requires extra costs, extra staffing, and time preparation, which hinders its large-scale development. Compared with other spinal surgical robots, the spinal surgical robot used in this study has the advantage of being able to support patients to perform surgery under local anesthesia and being less expensive.

## Data Availability

The original contributions presented in the study are included in the article/Supplementary Material, further inquiries can be directed to the corresponding author.
